# Thyrotoxic Periodic Paralysis: An Incidental Diagnosis!

**DOI:** 10.7759/cureus.7041

**Published:** 2020-02-19

**Authors:** Jennifer T Batch, Muhammad U Jahngir, Ismael Rodriguez

**Affiliations:** 1 Internal Medicine, Orange Park Medical Center, Orange Park, USA

**Keywords:** thyrotoxicity, hypokalemia, paralysis, na-k atpase, graves' disease

## Abstract

Thyrotoxic periodic paralysis is a rare presentation of thyrotoxicosis where the patient develops a transient motor deficit secondary to acute hypokalemia. The thyroid hormone augments gene transcription and post-transcriptional modification of Na-K ATPase, a cell membrane protein that regulates the electrical potential of the cell. Na-K ATPase increases active transport of potassium (K+) ions into the intracellular compartment causing hypokalemia without total body potassium deficit. Severe hypokalemia affects depolarization of the muscle cell membrane, clinically evidenced as paralysis. Other factors that may trigger hypokalemia and paralysis in the setting of hyperthyroidism include diet intake high in carbohydrates and salt, alcohol ingestion, trauma, infections, certain medication, and strenuous exercise. This rare but possible clinical presentation of thyrotoxicosis is significantly more predominant in males of Asian descent. We are reporting a case of a 44-year-old Asian-American male who presented to the emergency department with complaints of acute onset of bilateral lower extremity weakness. He had severe hypokalemia and was diagnosed with primary hyperthyroidism due to Graves’ disease.

## Introduction

Thyrotoxic periodic paralysis (TPP) is highlighted by the triad of thyrotoxicosis, muscle weakness and acute hypokalemia without total body potassium deficit [1]. TPP has a reported incidence of 1.8% - 1.9% among Asians, and 0.1% - 0.2% in the North American population [2]. Nevertheless, with globalization, mass immigration, and Westernization of food and lifestyle, TPP no longer has geographical boundaries [1]. Thyrotoxicity contributes to 16.6% - 32% of the hypokalemic paralysis and is 22 - 76 times more common in males [1-4]. We are reporting a case of TPP as an initial presentation of Graves' disease to reiterate the fact that patients with thyrotoxicosis can rarely present with reversible hypokalemia and muscle paralysis.

## Case presentation

**Figure 1:**
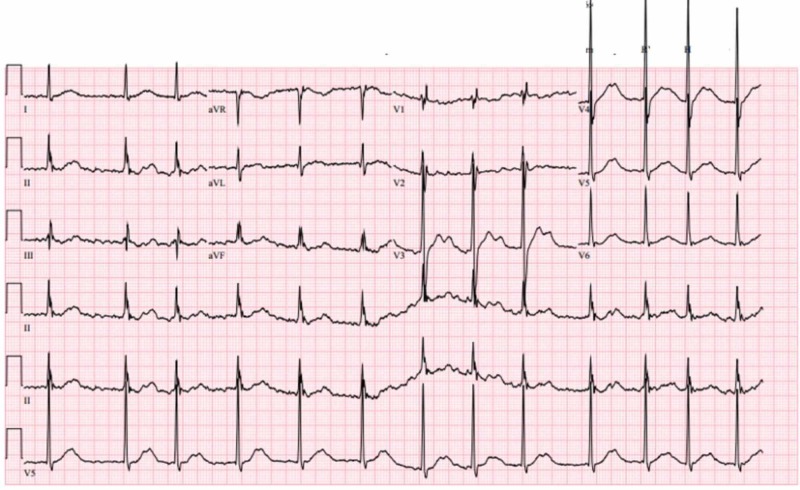
First electrocardiogram performed in the emergency department

A 44-year-old Vietnamese male patient with a past medical history of hypertension presented to the emergency department (ED) with complaints of the sudden onset of bilateral lower extremity weakness seven hours prior to the time of presentation. He was unable to stand or bear weight on his legs and it improved gradually by the time he arrived at the ED without any intervention. He denied head or neck trauma, paresthesias, or bowel/urinary incontinence. Two weeks prior, he had a dental infection treated with amoxicillin. He reported approximately 12 episodes of diarrhea in one day which resolved spontaneously. At presentation, his blood pressure was 191/73 mmHg and heart rate was 91 bpm with respiratory rate and body temperature within normal limits. Neurological examination revealed a motor deficit in both flexor and extensor muscle groups of the bilateral lower extremities with equal strength of 4/5 bilaterally and intact sensory function. An electrocardiogram (EKG) performed on admission revealed atrial fibrillation, along with an incomplete right bundle branch block and minimal voltage criteria of left ventricular hypertrophy (Figure 1). A comprehensive metabolic profile evidenced severe hypokalemia with a potassium level of 1.9 mEq/L and hyperglycemia with a glucose level of 221 mg/dl. Potassium was replaced via peroral route and hyperglycemia was dealt with short-acting insulin (per medium-dose sliding scale). Neuroimaging results were unremarkable. The atrial fibrillation was addressed with metoprolol succinate, 50 mg once a day. An echocardiogram showed preserved ejection fraction with normal valvular and systolic function.

**Figure 2:**
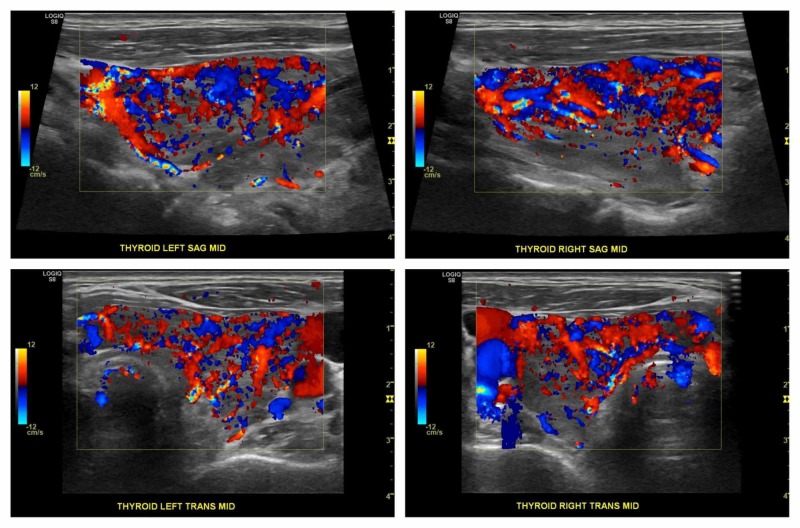
Ultrasound Doppler of the thyroid showing a hyperemic-appearing thyroid gland with a diffuse increase in blood flow in the left and right lobes of the gland Views in sagittal (SAG) (upper row) and transverse (TRANS) (lower row) planes

The atrial fibrillation on EKG (asymptomatic) led us to order a thyroid function profile for this patient, which favored primary hyperthyroidism with a suppressed thyroid-stimulating hormone (TSH) level of < 0.01 and high levels of free T4 and T3 of 5.85 ng/dl and 523 ng/dl, respectively. To estimate the clinical severity of thyrotoxicosis, a Wayne index score was calculated, which turned out to be 7 (4 for atrial fibrillation and 3 for heart rate (HR) > 90 bpm). A thyroid-stimulating immunoglobulin was ordered as a workup for the newly diagnosed hyperthyroidism and was found to be high at 19.8 IU/L (normal range: 0.00 - 0.55 IU/L). Thyroid ultrasound revealed a markedly heterogeneous hypoechoic and hyperemic-appearing thyroid gland with a diffuse increase in blood flow (Figure 2). These biochemical and ultrasonographic findings were consistent with Graves' disease. The patient was started on methimazole, 10 mg every eight hours, along with a beta-blocker, with the advice to follow-up in one week with his primary care physician and endocrinologist. Oral anticoagulants were not initiated due to a CHA2DS2-VASc score of 1.

Ten days later, the patient consulted for generalized body pruritus after gardening in an open backyard earlier that morning. He was given intravenous (IV) diphenhydramine, 25 mg, and had a complete resolution of his symptoms in the ED. The metabolic profile was within normal limits with a potassium of 4.3 mEq/L. He was discharged on oral diphenhydramine as needed and prednisone, 40 mg once daily for five days. On the following day, he presented once again in the late afternoon with complaints of a sudden onset bilateral lower extremity weakness in which it took him almost six hours to regain strength. The neurological exam demonstrated no motor or sensory deficits but he had hyperreflexia in all four limbs. Interestingly, a blood workup was normal, except for a potassium of 3.4 mEq/L and glucose of 277 mg/dl. The prednisone was stopped right after admission. He was discharged the following day when he was clinically asymptomatic and his electrolytes were within normal limits. He was counseled about his condition and was warned about the possible triggers for hypokalemia.

He was contacted six weeks after his hospital discharge. By that time, he had already been seen by his primary physician, almost a week before when his TSH level was within normal limits. He was compliant with methimazole, 10 mg three times daily, and metoprolol, 50 mg twice daily, and denied having similar complaints of muscle weakness since his hospital discharge.

## Discussion

TPP is a rare presentation of thyrotoxicosis and is most commonly associated with Graves’ disease [5]. Interestingly, only 17% of these patients had clinically overt thyrotoxicosis (Wayne index score > 19) during the first encounter in the ED, and most of them were diagnosed with thyrotoxicosis on their first presentation with hypokalemic paralysis [1, 6]. The mechanism of action of the thyroid hormone has been well-studied as it enters the cell and activates the thyroid response element (TRE) in the nucleus. This hormone-protein complex activates the Na-K ATPase gene transcription and post-transcriptional modification. Na-K ATPase maintains the resting membrane potential of the cell. The thyroid hormone eventually mediates increased expression of Na-K ATPase on the cell membrane, leading to an increase in adenosine triphosphate (ATP)-mediated influx of K+ ions into the intracellular compartment [7]. Apart from this direct anabolic effect of the thyroid hormone on protein synthesis, the activity of Na-K ATPase in skeletal muscles, liver, and kidney [7] is also enhanced indirectly by a T4-mediated increased release of insulin and response of catecholamine [2, 5, 8]. 

The extension of the above-mentioned mechanism can judiciously explain the gender predisposition of TPP in males. At a cellular level, the anabolic effect of testosterone on Na-K ATPase adds to the influence of thyrotoxicosis, in contrast to the decreased activity of Na-K ATPase in the presence of estrogen [2]. Androgens also increase total Na-K ATPase abundance in males by increasing the muscle mass and causing hypertrophy of myoblasts, thus increasing the muscle-to-body mass ratio [1].

Only 2% of the patients with thyrotoxicosis develop hypokalemia and muscle paralysis [1, 6], Therefore, other factors besides gender must come into play and predispose the patients to develop paralysis and hypokalemia. Inwardly rectifying potassium (Kir) channels mediate the K+ ion current across the cell membrane during repolarization. The Kir channel family is encoded by KCNJ genes [9]. The KCNJ18 gene encodes Kir 2.6, which is one of the seven Kir subfamilies [1-2, 9-10]. KCNJ18-related mutations have been found in up to 33% of the thyrotoxic patients with periodic paralysis [3, 11]. The KCNJ2 (Kir 2.1), and KCNJ12 (Kir 2.2) gene mutations are also associated with TPP [9]. Moreover, increased serum levels of catecholamine and insulin can also blunt the function of Kir channels [2]. It results in the negative balance of potassium ion concentration extracellularly. The resulting hypokalemia causes inactivation of voltage-gated sodium channels and finally leads to the paralysis of the motor unit [1]. A few human leukocyte antigen (HLA) genes are prevalent among Asian patients with Graves’ disease who developed TPP, but an independent association of HLA genes with TPP has yet to be discovered [9].

The possible precipitating factors of hypokalemia and paralysis in patients with underlying thyrotoxicosis include a diet high in carbohydrates and salt, alcohol ingestion, trauma, menses, infections, certain medication (e.g., steroids, diuretics, epinephrine, acetazolamide, and insulin), and strenuous exercise [1, 5]. We identified a viral gastrointestinal infection and the use of oral prednisone as a possible trigger of the respective episodes of paralysis in our patient. We acknowledge the fact that gastroenteritis had 'contributed' to hypokalemia in the setting of underlying undiagnosed primary hyperthyroidism. Other possibilities of paralysis (e.g., familial hypokalemic periodic paralysis, Guillain-Barré syndrome, myasthenia gravis, or spinal cord compression) were ruled out with history, physical examination, laboratory, and imaging studies in our patient [12].

**Figure 3:**
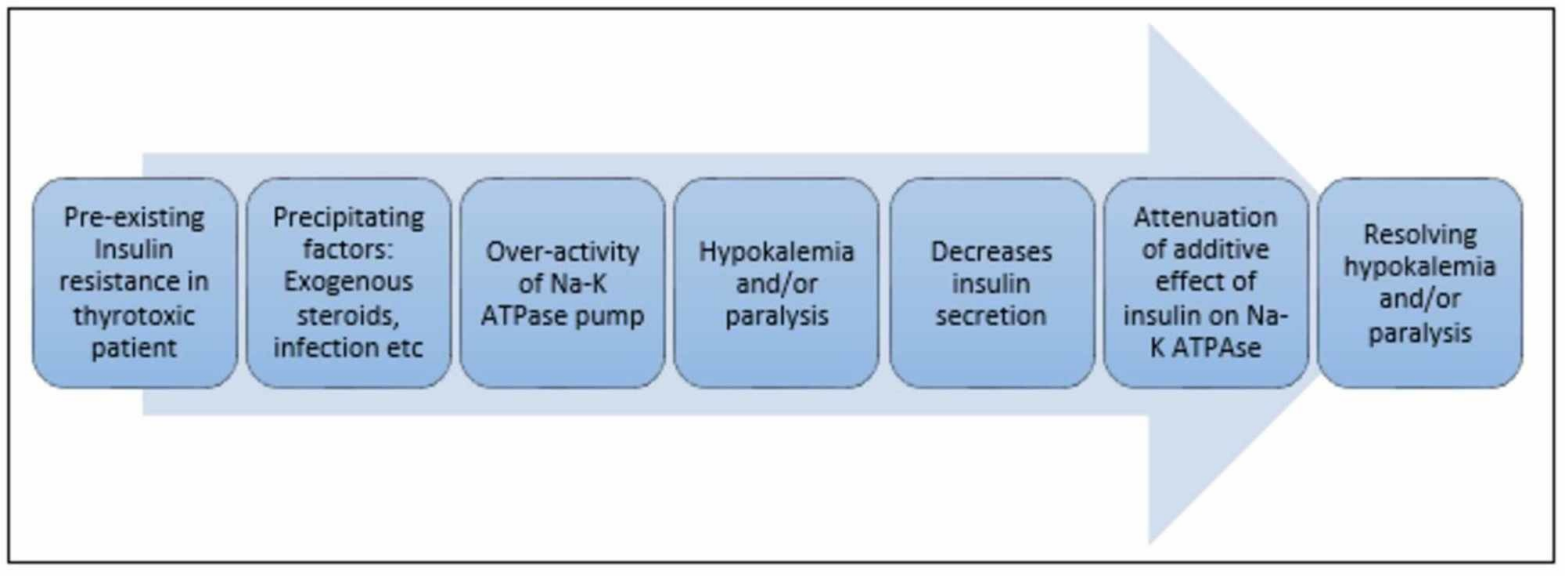
The algorithm for ‘self-limiting’ hypokalemia in patients with thyrotoxicosis

The severity of acute muscle paralysis correlates with the severity of the hypokalemia [1]. The algorithm for ‘self-limiting’ hypokalemia has been hypothesized in patients with thyrotoxicosis (Figure 3) [6]. It could be the probable reason for commonly seen ‘rebound hyperkalemia’ in those patients with TPP who received > 90 mEq of potassium in 24 hours as replacement therapy [1]. In most cases, less than 50 mEq of potassium chloride (KCl) is required for the acute management of TPP. If the patient is non-responsive to KCl, IV propranolol, 1 mg every 10 minutes, has also been proven effective in acute settings. It is found that prophylactic potassium replacement is not effective in these patients after the first reported episode of transient paralysis [2]. Beta-blockers, along with anti-thyroid medications, are better options for long-term management in these patients [2, 13]. 

Higher insulin resistance is reported in patients with TPP as compared to those with thyrotoxicosis who have never developed paralysis [1, 6]. Soonthronpun et al. studied low insulin sensitivity in patients with TPP but 90% of the subjects had a body mass index (BMI) of > 23 kg/m^2^ [6]. The compensatory hyperinsulinemia in the insulin-resistant population with thyrotoxicosis varies and is dependent on the capacity of functioning pancreatic beta-cells and thyroid-induced weight loss. Our patient had asymptomatic hyperglycemia on both presentations of paralytic episodes, with an HbA1c in the pre-diabetic range (6.1%), but our patient had normal weight for his height and gender (BMI: 22 kg/m^2^). Strict lifestyle modifications and repeat HbA1c in three months was advised on discharge.

TPP is an avoidable complication of hyperthyroidism and can rarely be fatal when it involves respiratory or bulbar muscles [14-15]. The main purpose of this case is to increase awareness of this condition. It is important for primary care providers to be vigilant about possible triggers for hypokalemia and paralysis in patients and to make sure that they are adherent to the definitive treatment of hyperthyroidism.

## Conclusions

Thyrotoxic periodic paralysis is a rare and completely reversible presentation of thyrotoxicosis if it is timely diagnosed and addressed adequately. Patient education about disease processes and possible trigger factors is necessary to prevent recurrent episodes of hypokalemia and paralysis in these patients.
